# Mother-daughter communication of sexual and reproductive health (SRH) matters and associated factors among sinhalese adolescent girls aged 14–19 years, in Sri Lanka

**DOI:** 10.1186/s12905-023-02617-4

**Published:** 2023-08-31

**Authors:** D. Mataraarachchi, Pathirana T.E. A, Mahesh Buddhika P.K, Vithana P.V.S. C

**Affiliations:** 1 Family Health Bureau, Ministry of Health, Colombo, Sri Lanka; 2 Base Hospital, Panadura, Sri Lanka; 3 Provincial Director of Health Services, Western Province Colombo, Sri Lanka

**Keywords:** Mother-daughter communication, Adolescent sexual and reproductive health

## Abstract

**Introduction:**

School-based and community-based sexuality education has not shown a significant impact on the sexual and reproductive health (SRH) outcomes of Sri Lankan adolescents. Parents, as the primary educators of adolescents, could potentially serve as better sources for providing individualized sexuality education to their children.

**Objective:**

To assess the sexual and reproductive health (SRH) topics discussed between mothers and daughters, barriers to communication, and associations of SRH communication among Sinhala adolescent girls aged 14–19 years in Sri Lanka.

**Methods:**

A descriptive cross-sectional study was conducted among a sample of 810 Sinhala adolescent girls using a pre-tested, self-administered questionnaire. Descriptive statistics pertaining to mother-daughter communication in SRH matters were presented using frequencies and percentages. Bivariate analysis was performed to evaluate the association of selected socio-demographic factors with mother-daughter SRH communication, while binary logistic analysis was used to assess the independent association of chosen demographic factors with mother-daughter SRH communication.

**Results:**

The majority of the respondents (67.1%, n = 540) indicated a willingness to discuss SRH matters with their mothers. For 78.2% (n = 632) of the respondents, the mother was the preferred source of SRH information. Common topics discussed between the mothers and daughters included issues related to menstruation (88.4%, n = 701) and maintaining personal boundaries (94%, n = 718), whereas less discussed topics included homosexuality (21%, n = 166), and preventing unwanted pregnancy (38.6%, n = 305). Cultural barriers, fear of mothers’ reactions, and mothers’ lack of confidence in responding to their daughter’s SRH matters were the main barriers to discussing SRH topics with mothers. Adolescent girls’ age, and family income level were significantly associated with mother-daughter SRH communication.

**Conclusions and recommendations:**

Although a majority of adolescent girls preferred to share their sexual health concerns with their mothers, a notable barrier was the lack of confidence from the mother’s side. Furthermore, the scope of mother-daughter sexual communication in this study was largely limited to less sensitive topics. In light of these findings, it is suggested that interventions targeted at parents be developed alongside school-based sex education for adolescents. These interventions should aim to enhance parents’ knowledge and skills in discussing sexual health matters with their adolescent children.

**Supplementary Information:**

The online version contains supplementary material available at 10.1186/s12905-023-02617-4.

## Introduction

More than half of the world’s adolescent population hails from Asia, with South Asia alone accounting for 344 million [1]. In Sri Lanka, adolescents constitute 23.2% or 4.64 million of the total population.

Adolescence encompasses the period from the onset of puberty until a child reaches adulthood. During this phase, adolescents undergo substantial physical, mental, and social changes that can expose them to high-risk behaviors with potential adverse impacts [2]. Emerging sexual and romantic interests play a significant role during adolescence [3]. There is an increased inclination towards intimate relationships and sexual exploration during this time [4]. However, the relative immaturity of adolescents compared to adults, in terms of physical, cognitive, and emotional development heightens the risk of negative sexual outcomes [5]. The rising prevalence of premarital sex, coupled with inadequate awareness and skills related to sexual and reproductive health (SRH) has led to a higher incidence of sexually transmitted infections (STIs), unintended pregnancies, abortions, and instances of sexual violence and coercion among adolescents in the region [6].

Recent evidence indicates a noteworthy increase in the average age of marriage among Sri Lankan women over the past century, which has correspondingly led to higher rates of premarital sex among Sri Lankan females [7]. Reports from previous years highlight a consistent adolescent fertility rate of 31 and 30 per 1000 women aged 15–19 years over a span of 40–45 years [8], indicating a lack of notable progress in the field of adolescent sexual and reproductive health within the country. In 2019, the national teenage pregnancy rate stood at 4.4% [8], encompassing 16,708 reported cases among adolescents under the age of 19.

Research has identified a significant lack of knowledge about SRH as a key contributor to the growing burden of SRH issues within the country [9, 10]. Despite national and district level efforts to enhance SRH indicators among Sri Lankan adolescents and youth, the literature reveals that these interventions have had limited success in improving SRH knowledge, attitudes, and practices among youth [10, 11]. Although SRH education has been integrated into the school curriculum for over three decades, minimal progress has been observed in terms of SRH knowledge, attitudes, and behaviors among youth in the country [10, 12, 13]. Studies demonstrate that many Sri Lankan youth possess limited SRH knowledge, leading to heightened risks of problematic behavior [14]. A mere 58% of students expressed satisfaction with the existing school-based SRH education in Sri Lanka [15]. A study conducted among youth in Nuwaraeliya, identified schoolteachers as the least reliable source of sexual health information [16]. Moreover, the national youth health survey revealed that Sri Lankan youth perceived schoolteachers as an inadequate source of information on puberty, contraception, or other sexual health-related matters [13]. Furthermore, a recent study among youth trainees at national youth training centers in Sri Lanka highlighted subpar knowledge, attitudes, and practices regarding sexual and reproductive health [11]. These factors may contribute to the dissatisfaction of students regarding school-based SRH education.

Research indicates that beyond formal school based sexual education, adolescents receive sexual health information from diverse sources such as books, magazines, parents, teachers, friends, siblings, and the internet. International literature underscores the role of parents as a primary source of SRH information for their children [17, 18]. The capacity of the parent to provide personalized sexual health information tailored to their child’s religious, cultural, and family background positions them as ideal source of SRH guidance. Furthermore, global evidence shows that parent-adolescent SRH communication can mitigate unhealthy SRH practices among adolescents and enhance their overall well-being [19, 20].

The National Youth Health Survey reveals that mothers were sources of puberty related information for 75% of the adolescent girls in Sri Lanka [13]. Additionally, 29% of girls received information from parents about issues like teenage pregnancy [13]. However, no comprehensive study has investigated the dynamics of parent-adolescent sexual health communication within the Sri Lankan context, particularly adolescent’s perspectives on discussing sexual health matters with their parents. International studies indicate that family-centered sex education effectively enhances adolescents’ knowledge, attitudes, and SRH practices. Adapting or designing such interventions in Sri Lanka necessitates an assessment of mother-daughter communication patterns on SRH among Sinhala adolescent girls. This study aims to explore existing mother-daughter sex communication patterns within the present study setting. The study exclusively focuses on mothers’ communication with their daughters regarding SRH, as previous literature highlights gender-based disparities in parent-adolescent sexual health communication, wherein male children’s communication with their mothers about sexual and reproductive health matters differs from that of females [21]. The study encompasses adolescent girls aged 14–19 years, given that the current sexuality education in schools typically commences in grades 8–9 (13–14 year age group), and this age group is particularly susceptible to risky sexual behaviors. The choice of Kalutara District as the study setting, is due to its significant population size, making findings potentially generalizable to the overall population of Sri Lanka.

## Methodology

### Design and setting of the study

A descriptive cross-sectional study was carried out in Kalutara district, Sri Lanka from November 2019 to April 2020. Kalutara district is having the second highest population in Sri Lanka, with Sinhala being the predominant ethnic group.

### Study participants

Data were collected from 810 female adolescents aged 14–19 years belonging to Sinhalese families residing in Kalutara District.

### Sample size calculation

The sample size was calculated using the single population proportion formula [19] applying the anticipated proportion of mother-daughter SRH communication of 50% as there were no published studies. The design effect was taken as 1.9 after adjusting for the homogeneity of clusters. With a 10% non-response rate, the final sample size was 810.

### Sampling method

Adolescents were selected using probability proportionate to size, multistage, and cluster sampling techniques. A cluster of 10 was taken based on the number of adolescent girls aged 14–19 years that could be sampled during a day from a PHM area.

### Inclusion and exclusion criteria

Only the adolescent girls who were living with their biological mothers at least two days per week were included in the study sample. Illiterate adolescents and girls who were having communication disabilities and whose mothers were having communication disabilities were excluded. Further, adolescents who were married or living with a partner were excluded from the study.

### Data collection

Data collection was carried out using a self-administered questionnaire by three female university students who were trained by the principal investigator. Since the globally available questionnaires contained questions that did not match the cultural and religious background of the present study setting and did not cover all objectives in the present study, a new questionnaire was developed after an extensive literature search and expert opinion.

The questionnaire was assessed for face and content validity and pre-tested among a group of 30 adolescent girls in the same age group. All the composite scales in the questionnaire had an internal consistency of > 0.7 Cronbach’s alpha value and test-retest reliability of > 0.8 Cohen’s Kappa value.

Data collectors were trained on getting consent from the parents and adolescents, communicating with the youth, and ensuring the information’s privacy and confidentiality. Data collection was carried out during the weekend, on public holidays, or after school hours. Data collectors visited each household in the given cluster looking for adolescent girls in the respective age group. Data collection was done out of their households to minimize the influence of the family members. Those who consented to participate in the study were gathered at a common place such as the PHM office, community hall, or one of the households within the area, after obtaining consent from parents and assent/consent from adolescents. Parents were explained and requested not to be present during the time of responding to the questionnaire.

The adolescent girls who were not available in their households during data collection and the adolescent girls whose parent/guardian was not available were not included in the study.

### Data Analysis

All data were coded and entered into a database that was created using a standard statistical package, IBM SPSS version 25. The demographic and social characteristics of the respondents were analyzed using descriptive statistics. Categorical data such as religion, family income level, adolescent girls’ living status, presence of siblings at home, education level of the mother and the daughter, and mother’s employment status, were described in numbers and percentages. Descriptive statistics related to mother-daughter communication of SRH issues were studied using frequency distributions and presented in numbers and percentages.

A scoring system was developed to assign scores to the level of mother-daughter communication of SRH topics. The scoring system was developed with the advice of the same panel of experts who were involved in questionnaire development. The scores were assigned in a manner where the most favorable response was given the highest and the least favorable, the lowest [22]. Since we could not come across any literature on a well-validated cut-off value for mother-daughter SRH communication, the scores were categorized around the 60th percentile. This was considered after going through previous literature and consensus generation among the experts in the field of research and adolescent health. Consensus generation among experts regarding the scoring system and the cut-off was done at the time of questionnaire development using the Modified Delphi process through the electronic mail system. The 60th percentile is a percentile value that is often used in research and statistics [23]. Furthermore, it is seen as a point that balances data distribution or strikes a balance between sensitivity and specificity. During the preliminary analysis, sensitivity analysis was conducted by varying the cut-off percentile (50th and 70th) and it was confirmed that the 60th percentile cut-off aligns reasonably well with the present study findings.

The cut-off was utilized only to determine the associations of selected demographic factors.

Complete Case Analysis was performed where the missing data were discarded and only the complete observations were analyzed. This was performed assuming that the missing was at random. The use of Complete Case Analysis helped to ascertain the true effect of the event since the data reflect only the cases for which all necessary information is available [24]. Furthermore, since the proportion of missing data in the present study was minimal (less than 5%), the use of complete case analysis provided an unbiased estimate.

## Results

The mean age of the respondents was 15.3 years with a standard deviation of 1.7. Of the 810 respondents, all were Sinhalese, while 94.7% (n = 767) were Buddhists. Nearly 89% of the adolescent girls in the sample were schooling (Table 1).

**Table 1:** Demographic characteristics of the participants

Variable	Frequency (N)	Percentage %
**Age in Years (n = 807)**		
14	318	39.5
15	179	22.2
16	88	10.9
17	125	15.5
18	63	7.8
19	34	4.1
**Ethnicity (n = 810)**		
Sinhalese	810	100.0
Other	0	0.0
**Religion (n = 810)**		
Buddhist	767	94.7
Christian	28	3.5
Catholic	15	1.8
**Status of schooling (n = 802)**		
Schooling	712	88.8
Engaged in higher studies	51	6.3
Following the vocational training course	11	1.4
Employed	8	1.0
Not studying nor employed	20	2.5
**Monthly Family Income (n = 786)**		
Less than Rs.20,000	120	15.3
Rs.20,000-Rs.50,000	238	30.3
>Rs. 50,000-Rs. 100,000	98	12.4
More than Rs.100,000	24	3.0
Do not know	306	39.0
**Presence of Siblings (n = 800)**		
Yes	743	92.9
No	57	7.1

### Adolescent girl perceived usefulness of mother-daughter SRH communication

In the opinion of 67.1% (n = 540) of adolescent girls, it was a good decision to discuss SRH-related matters with mothers. A greater proportion of the girls (86.4%, n = 698) thought that talking to their mothers about SRH-related matters will help to protect themselves from sexual harm in the future. Nearly 27% (n = 207) of the girls were doubtful about their mothers’ ability to answer their sexual health matters (Table 2).

**Table 2:** Adolescent girl perceived usefulness of mother-daughter SRH communication

Item	Yes		No		Don’t Know	
	N	%	N	%	N	%
Do you think discussing sex-related matters with your mother is a good decision(n = 805)	540	67.1	265	32.9	-	-
Do you think it would be helpful to talk to your mother about sex-related matters to protect yourself from any such issues that might happen in the future (n = 808)	698	86.4	8	1.0	102	12.6
Do you think your mother can answer your SRH-related matters (n = 779)	570	73.2	22	2.8	187	24.0
Are you satisfied with your mother’s answers to your questions on sexual health (n = 792)	557	70.3	76	9.6	159	20.1

*SRH- Sexual and Reproductive health

### Sexual and reproductive health topics discussed between the mothers and daughters

A higher proportion of mothers in the sample (94%, n = 744) had talked to their adolescent girls about keeping body limits. Many (88.4%, n = 701) had discussions with their daughters about menstrual-related issues, while 72.6%, n = 574) had discussions on preventing sexual violence.

More than half (57.6%, n = 455) of the adolescent girls in the sample had discussed love, relationships, and marriages with their mothers. However, not many (38.7%, n = 305) had discussed the consequences of premarital sex, preventing unwanted pregnancy (38%, n = 305), or about sexually transmitted diseases (40.1%, n = 317). Table 3 indicates SRH topics discussed between mothers and adolescent daughters in the present study setting.

**Table 3:** Sexual and reproductive health topics discussed between the mothers and daughters

Variable	Yes		No	
	N	%	N	%
**Has your mother ever talked to you about;**				
Keeping body limits (n = 792)	744	94.0	48	6.0
Problems related to the menstrual cycle and puberty (n = 793)	701	88.4	92	11.6
How to protect yourself from sexual violence (n = 791)	574	72.6	217	27.4
Love, relationships, and marriages (n = 790)	455	57.6	335	42.4
Sexually transmitted infection (n = 790)	317	40.1	473	59.9
Consequences of premarital sex (n = 788)	305	38.7	483	61.3
Preventing unwanted pregnancy (n = 790)	305	38.6	485	61.4
Pregnancy and conception (n = 780)	271	34.7	509	65.3
Homosexuality (n = 790)	166	21.0	624	79.0

### Adolescent perceived barriers to discussing sex-related matters with mothers

The main reason why the girls in the study refrained from discussing sexual health matters with their mothers was that they feared the negative perception they may receive from the mother (56.3%, n = 456). A significant proportion (46.8%, n = 379) was also worried about the cultural restraints over discussing sexual problems in open n. Other barriers to discussing SRH matters with mothers are listed in Table 4.

**Table 4:** Adolescent perceived barriers to discussing sex-related matters with mothers

Variable	Yes		No	
	N	%	N	%
I’m scared about how my mother will perceive it (n = 810)	456	56.3	354	43.7
Open discussion on sex-related matters is not culturally acceptable(n = 810)	379	46.8	431	53.2
My mother can’t respond to my sexual issues (n = 810)	149	18.4	661	81.6
My mother is too busy. She can’t find time to discuss it with me (n = 810)	135	16.7	675	83.3
My mother doesn’t understand my needs (n = 808)	43	5.3	765	94.7
I’m embarrassed over discussing sex-related matters with parents (n = 810)	36	4.4	774	95.6
My mother doesn’t want to share sex-related information with me (n = 809)	36	4.4	773	95.6

### Current sources of sexual Health information to adolescent girls

Mother was a source of sexual health information for many adolescent girls (n = 687, 84.8%), followed by teachers (n = 570, 70.3%). Furthermore, 69.5% (n = 563) of the girls reported friends as a source of sexual health information. Sources of sexual health information of the girls in the present study sample are shown in Table 5.

**Table 5:** Current sources of sexual health information to adolescent girls

Source of sexual health information (n = 810)	Yes		No	
	Frequency (N)	Percentage (%)	Frequency (N)	Percentage (%)
Mother	687	84.8	123	15.2
Teachers	570	70.3	240	29.7
Friends	563	69.5	247	30.5
Books and Journals	434	53.6	376	46.4
Television	338	41.7	472	58.3
Health officers	271	33.5	539	66.5
Siblings	178	22.0	632	78.0
Internet	121	14.9	689	85.1
Father	54	6.7	756	93.3

### Associations of mother-daughter sexual and reproductive health communication with selected demographic factors

The binary logistic regression model with two predictor variables showed that age and family income were significantly associated with mother-daughter SRH communication (X^2^ = 51.33, df = 6, P < 0.01). In the present study sample, mother’s communication of SRH matters was 2.8 times (AOR = 2.8, 95% CI: 1.9–4.2; p < 0.001) more satisfactory as perceived by older girls above 15 years, compared to younger girls below 15 years, when adjusted for other confounding variables; family income level, schooling status, religion, mother’s education level, and mothers’ employment status [Table 6].

Similarly, mother-adolescent daughter SRH communication was 2.4 times (AOR = 2.4, 95% CI: 1.5–3.8; p < 0.001) high as perceived by adolescent girls from high-income families compared to those from low-income families, when adjusted for confounding variables; age, family income, religion, schooling status, mothers’ education level, and mothers’ employment status [Table 6].

However, mother-daughter sexual communication was not significantly associated with adolescent girls’ schooling status, religion, mothers’ highest education level, or mothers’ employment status.

**Table 6:** Association of Mother-Daughter Sexual and Reproductive Health Communication with Selected Demographic Factors

Characteristic	Mother-daughter communication of sex				Total		Unadjusted OR	Adjusted OR
	Satisfactory		Unsatisfactory		No	%	(95%CI)	(95%CI)
	N	%	No	%				
**Age of the adolescent girl (n = 807)**								
< 15yrs	193	62.2	117	37.8	310	100	3.0	2.8
≥ 15yrs^#^	177	35.6	320	64.4	497	100	(2.2–4.4)	(1.9–4.2)
**Family income (n = 480)**								
< Rs.50,000	148	41.3	210	58.7	358	100	2.3	2.4
≥ Rs. 50,000^#^	76	62.3	46	37.7	122	100	(1.5–3.5)	(1.5–3.8)
**Schooling status (n = 802)**								
Out of school	52	57.6	38	42.4	90	100	1.7	1.4
Schooling^#^	315	44.2	397	55.8	712	100	(1.1–2.6)	(0.7–2.7)
**Religion (n = 808)**								
Non-Buddhist	9	22.0	32	78.0	41	100	3.1	1.2
Buddhist^#^	362	47.2	405	52.8	767	100	(1.4–6.7)	(0.6–1.6)
**Mothers’ highest Education (n = 762)**								
Below GCE.O/L	37	39.4	57	60.6	94	100	1.4	1.8
G.C.E.O/L &above^#^	320	47.9	348	52.1	668	100	(0.9–2.2)	(0.9–3.3)
**Mothers’ Employment status(n = 810)**								
Unemployed	273	44.0	347	56.0	620	100	1.4	1.4
Employed^#^	98	51.6	92	48.4	190	100	(1.0–2.0)	(0.9–2.2)

**Multi-variable logistic regression model, significance at P < 0.05 level

# Reference category

## Discussion

The study’s findings indicate that a majority of adolescent girls within the present study setting exhibited a positive attitude towards discussing SRH matters with their mothers. Topics related to menstrual health, setting personal boundaries, and preventing sexual violence were among the most frequently discussed subjects between mothers and daughters. Barriers to these conversations included cultural factors, concerns about parental perception, and mothers’ inability to address adolescent SRH matters. The analysis revealed significant associations between adolescent girls’ age and family income levels with mother-daughter SRH communication.

The current study’s findings are consistent with prior research, suggesting that mothers remain a preferred source of SRH information for adolescent girls in this setting [12]. A substantial portion of the girls believed that discussing sex-related matters with their mothers could contribute to safeguarding themselves against potential sexual harm in the future. A qualitative study conducted among adolescent girls in the MOH area of Beruwala similarly indicated a preference for maternal advice on sexual health matters [14]. However, in contrast to the above study, which reported that many adolescent girls contacted their friends for sexual health matters [12], the mother was the main source of sexual health information for the girls in the present study.

Similar to the present findings, a community-based study carried out among pregnant adolescents in three districts in Sri Lanka [25] indicated that girls had a good level of freedom to discuss matters related to growth, puberty, and sexuality. Moreover, findings indicated that pregnancy and conception, safe sex, and homosexuality were among the unpopular topics to discuss between mothers and teenage daughters in the present study setting. This finding is consistent with research conducted in South Asian countries with similar cultural backgrounds [26]. Zakaria’s work in Bangladesh [26] demonstrated that menstruation-related issues were commonly discussed between mothers and daughters (67%), whereas discussions about sexual violence and abuse prevention were less prevalent (14%).

Culture significantly shapes attitudes, beliefs, and behaviors, including parent-child relationships [27]. The study’s findings suggest that concerns about the sexual security and chastity of female offspring predominantly drive mother-daughter sexual communication in Sri Lanka, mirroring trends in other South Asian countries. Conversations tend to focus more on body protection rather than on topics such as safe sex, pregnancy, and conception. Similarly, a study conducted in Indonesia found that body changes during puberty are the major content of mother-daughter sexual communication, and they tend to avoid discussing other SRH topics [28]. In contrast to the above findings from Asian settings, a cross-sectional study carried out among 557 parents in Australia reported that the most useful topics to be discussed with an adolescent girl were the prevention of sexual abuse and encouraging a positive body image [29]. Moreover, a study conducted in Uganda found that mothers actively encouraged condom use among their daughters [30].

These findings underscore the contrast in sociocultural values between Eastern and Western settings. Parents in Eastern countries often prioritize the protection of their daughters’ chastity, while Western cultures tend to emphasize bolstering their daughters’ self-confidence. The findings further highlight the fact that girls in the present study setting were neither prepared to prevent an unexpected pregnancy nor taught about the process of pregnancy and conception. Existing literature advocates transitioning from abstinence-only sexual health education to comprehensive sexuality education for adolescents, as the former leaves minors ill-equipped to manage their sexuality [31].

Approximately one-fifth of the girls surveyed expressed uncertainty about their mothers’ capacity to address their SRH queries. Moreover, around one-third of the girls reported being either dissatisfied or uncertain about the adequacy of their mothers’ responses regarding SRH matters. This indicates the necessity for parent-oriented interventions aimed at enhancing mothers’ ability to engage in conversations on SRH issues, encompassing improvements in both knowledge and communication skills.

The study identified the fear of mothers’ judgment as a central barrier to SRH discussions between mothers and adolescent girls. This finding aligns with a US study demonstrating adolescents’ hesitancy to engage in sexual conversations with their mothers due to fear of parental perceptions [32], as well as a Nigerian study in which parents perceived children initiating such discussions as either sexually active or planning to be [33]. Similarly, a study conducted among schoolchildren in New York City [34] disclosed that both adolescent boys and girls feared posing sex-related questions, fearing that it might trigger unnecessary parental suspicion. These findings highlight the need to reshape parents’ attitudes toward SRH communication.

Cultural barriers and mothers’ limitations in addressing adolescent SRH issues were identified as barriers to sexual health discussions between mothers and daughters in the current study. A comparative analysis exploring mother-daughter sexual communication differences among white, black, Latin, and Asian mothers indicated that non-white individuals experienced greater discomfort discussing sexual health topics with their children [35]. These findings advocate for public health awareness initiatives aimed at enhancing knowledge and attitudes surrounding SRH within this context.

The study also found that older adolescents perceived their sexual health communication with mothers to be more satisfactory than younger counterparts. This trend aligns with several studies conducted in Asia and similar settings, indicating that parents often prefer to initiate sexual discussions when children reach around 15 years of age, a period when most have already undergone puberty and acquired some prior knowledge about sexuality [36, 37]. A Nigerian cross-sectional study regarding timing and initiation of sexuality discussions revealed that parents typically prefer to broach the topic with children only after they enter puberty [37]. Furthermore, a systematic review evaluating parental preferences for adolescent sexuality education indicated that parents often perceive their children as too young to receive SRH information, which in turn hampers open dialogue [38]. In contrast to these findings in Asian settings, a review of literature from Western cultures suggested that mothers welcomed initiating sexual education for their children at a younger age, such as during elementary school or even earlier [39]. A similar study in the West proposed that pre-adolescence, typically around 11–14 years old, is an opportune phase for mothers to commence sexual discussions with their children, as children tend to be curious and undergo physical and psychological changes during this period [40, 41]. Additionally, research has indicated that late adolescence can pose challenges for sexual conversations due to teenagers’ desire for independence and separation from their family unit.

Furthermore, adolescents from higher-income families were more likely to report satisfactory mother-daughter sexual communication than those from lower-income backgrounds. Similar findings emerged from a previous study in Sub-Saharan Africa [42], where families with higher socioeconomic status demonstrated more prevalent sexual discussions between parents and adolescent children.

### Strengths and weaknesses of the study

The current study was the first to assess the parent-adolescent sexual communication pattern within this study’s specific setting, introducing a novel argument for SRH advocacy. A community sample was obtained through a well-designed sampling process that ensured the unbiased inclusion of adolescent girls from throughout the Kalutara district. The district holds the second highest population among all districts in the country, encompassing residents from urban, rural, and estate sectors. The use of self-reported data, data collectors being youth, and conducting data collection at a place away from their households served to assure participants of information confidentiality, thereby enhancing the reliability of the received data.

The study was confined to Sinhala mother-daughter pairs, thereby enhancing the applicability of the study findings to the Sinhala community in Sri Lanka. However, the generalization of study findings to other ethnic groups within the country should be approached cautiously, due to potential attitudinal, and sociocultural disparities as indicated by prior literature [33]. Data collectors conducted a single household visit, without subsequent revisits. This approach might have led to an under-representation of specific age groups such as those attending Ordinary Level (O/L) and Advanced Level (A/L) tuition classes, constituting a study limitation.

Limitation of the study included reporting of family income level by the adolescent participants, raising concerns about data accuracy. Merely 6% of the daughters in the study sample reported their fathers as sources of sexual health information. However, the questionnaire failed to gather information about the percentage of study participants residing with both parents or single parents, warranting cautious interpretation of this finding. In addition, the cut-off value utilized to differentiate mother-daughter pairs with satisfactory SRH communication from those with unsatisfactory SRH communication, was established based solely on prior literature and expert consensus, lacking a comprehensive validation process. This could introduced uncertainty into the study’s conclusions. Nevertheless, since this cut-off was exclusively employed to ascertain the socio-demographic link to satisfactory mother-daughter SRH communication within this study setting, the potential negative impact of using a non-validated cut-off might be minimal.

### Public health implications of the study findings

This study offers valuable insights into the prevailing context of mother-adolescent sexual communication within the study setting. The findings underscore that adolescent girls in this context possess an inclination to share SRH information with their mothers. However, this inclination is often hindered by a range of barriers, including apprehension about their mother’s perception, uncertainty regarding their mother’s ability to address SRH-related queries, and adherence to cultural taboos surrounding the topic.

The adolescent girl’s desire to have their mother as a source of sexual health information serves as a promising opportunity when planning public health programs aimed at enhancing SRH awareness among children and adolescents. Nonetheless, the study highlights the necessity for fostering positive shifts in mothers’ knowledge and attitudes concerning sexuality education of adolescents. Additionally, it underscores the importance of equipping parents with the skills to effectively engage in discussions about SRH matters with their children. These imperatives currently stand as prerequisites for promoting family-centered sexuality education within the study setting.

To address these requirements, it becomes imperative to strategize and implement school-based and community–based interventions that are tailored to engage parents. These interventions should aim at improving mother’s knowledge and skills in adolescent SRH, thereby facilitating the creation of a supportive environment for open and informed discussions about SRH within families.

## Conclusion

The majority of the girls in our study exhibited a willingness to engage in discussions about SRH with their mothers. However, these conversations between mothers and daughters predominantly revolved around less sensitive topics such as issues related to menstruation, keeping body limits, and preventing sexual violence. However, it became apparent that certain topics such as love, relationships, marriages, pregnancy, or contraception were less openly discussed within the family environment. This discrepancy can be attributed to cultural barriers, concerns regarding parental perception, and mothers’ potential unease in responding to adolescent SRH inquiries.

The study also revealed that discussions about SRH tended to take place more openly between mothers and older daughters, while these discussions were relatively limited with younger daughters. Additionally, findings indicated that adolescent girls hailing from higher-income families perceived a greater frequency of sexual health discussions with their mothers in comparison to those from lower-income backgrounds.

### Recommendations

Policymakers need to consider parents as a primary source of sexual health information for adolescent girls. An integrated approach where the family and community play a facilitating role for school-based SRH education is an effective way of delivering SRH education to Sri Lankan adolescents. In this regard, the implementation of parent-oriented awareness campaigns and skill-building sessions in conjunction with school-based sexual health education programs is highly recommended.

### Suggestions for future research

Given that the present study exclusively focused on Sinhala mothers and daughters, it is imperative that research be conducted to encompass all ethnic groups and religious affiliations. This approach will enable comprehensive understanding of the distinct needs within various communities residing in the country.

Furthermore, the study’s scope was confined solely to mother-daughter pairs. Future research should extend its exploration to encompass the pattern of SRH communication between mothers and sons, fathers and daughters, and fathers and sons. This broader investigation will help to identify the optimal parent figure for effectively conveying health messages to adolescents.

The study findings revealed enhanced sexual discussions between mothers and daughters from higher income families. Future research should be designed to provide clarity on this observation. Additionally, there is a compelling need for future research to utilize a meticulously validated cut-off value when discerning mother-daughter dyads with satisfactory and unsatisfactory SRH communication.

To enhance study’s acceptance, it was conducted among late adolescents. Nevertheless, evidence underscores the importance of initiating parent-adolescent communication regarding sexual matters during early adolescence, prior to the initiation of sexual activity, in order to yield optimum outcomes [43]. Consequently, there exists a need for forthcoming research to pinpoint the most suitable age for initiating sexual discussions with adolescents in Sri Lanka.

The study targeted only one daughter from a family. However, it is plausible that the same mother communicate differently with two or more daughters. Further research should examine the pattern of SRH communication of the mothers with two or more daughters in the same family, while also exploring the adolescent factors that might contribute to maternal communication of SRH matters.

Furthermore, it is recommended to undertake dyadic research involving both mothers and adolescent daughters. This approach would ascertain whether disparities exist in the perception of SRH communication between the two groups.

### Electronic supplementary material

Below is the link to the electronic supplementary material.

Additional File 1: Annexure 1

Additional File 2: About the topic

## Data Availability

The datasets used and/or analyzed during the current study are available from the corresponding author upon reasonable request.
