# Supplementary material for: Mother-daughter communication of sexual and reproductive health (SRH) matters and associated factors among sinhalese adolescent girls aged 14–19 years, in Sri Lanka
Source: BMC Womens Health. 2023 Aug 31;23:461. doi: 10.1186/s12905-023-02617-4 (PMC10472576; doi:10.1186/s12905-023-02617-4)
Supplement: Supplementary file 1 — Additional File 1: Annexure 1 [file 12905_2023_2617_MOESM1_ESM.pdf]

Annexure 1

Confidential

Self-administered Questionnaire to assess the existing pattern of mother-daughter communication on sexual and reproductive health matters among adolescents aged 14-18 years in Kalutara district

Details

Serial Number [ ]

Date of Survey …..../……./…….. (Day/Month/Year)

Check the Eligibility

		Yes	No
1	Be able to understand Sinhala language		
2	Living with biological mother in the same house-hold at least for 2 days per week during the past 6 months		
3	Having a mother who does not have any communication difficulties		

If the answers to above questions are 'Yes', proceed after obtaining consent.

Socio-Demographic information

1. Sex: F/M
2. Age: …………
3. Ethnicity: 1. Sinhala 2. Tamil 3. Muslim 4. Burgher
   5. Other (specify)……………….
4. Religion: 1. Buddhist 2. Catholic 3. Cristian 4. Hindu
   5. Islam 6. Other (specify)……………….
5. What do you do now? 1. Going to school
   2. I just stay in home
   3. Engaged in higher studies
   4. Engage in vocational or job training
   5. Follow a course (English/computer)
   6. Doing a full-time job
   7. Doing a part-time job
   8. Other ………………………

If you are going to school go to Q6, If you are not schooling, go Q7

6. If you are schooling, what is the grade that you are in at present? ……………………..

7. If you are not schooling at the time of completing the questionnaire, what are your highest educational qualifications?

   a) I have not attended to school ever

   b) Upto grade 1-5

   c) Upto grade 6-8

   d) Up to grade 9-11

   e) Passed GCE O/L exam

   f) Up to A/L

Living Conditions

8. Is your mother living with you in the same household?

   a) Yes b) No

If no, go to Q9. If yes, go to Q12

9. If no, why?
   a) She is working in a separate place in the country
   b) My mother is currently working in a foreign country
   c) My mother is divorced/separated from my family
   d) Other (specify)………………………….

If your answer to Q9 was No, go to Q12

10. If you are living with your mother, for how many days per week you live with her in the same household?
    a) Almost all days per week
    b) More than 4 days per week
    c) Less than 2 days per week
    d) Other (Specify) ………………………….

11. Do you have sisters or brothers? a) Yes b) No

    No. of sisters ……………….

    No. of brothers ……………

Socio-economic background

12. What are the education qualification of your mother:
    a) Less than Grade 5
    b) GCE O/L pass
    c) GCE A/L Pass
    d) Diploma
    e) Degree or above
    f) Post graduate
13. Average monthly income of the family
    a) Rs.20,000 or less
    b) Rs.20,000 – Rs.50,000
    c) Rs.50,000- Rs.100,000
    d) Rs.100,000 or above
    e) I do not know

14. Residence: a) Urban b) Rural

15. What is your father's occupation? (If the father is dead, tell about the occupation he was involved when he was alive)

    ………………………………………………………………………………………………..

16. What is your mother's occupation? (if mother is dead, tell about her occupation when she was alive)

    ………………………………………………………………………………………………..

Mother-daughter relationship

19. Who is your closest feeling parent?

a) Mother b) Father c) Both d) None of the above

20. How do you think of your mother?

	Strongly agree	Agree	Disagree	Strongly agree
I think highly of my mother				
She is the person I want to be like				
I really enjoy spending time with her				

21. Perceived love and trust from mother

	True	False
a) My mother enjoys spending time with me		
b) My mother shows me that she loves me		
c) My mother shows me that she trusts me		

22. Perceived supportiveness of the mother

How often does she praise you for doing well	Never	Sometimes	Most of the times	All the time
How often does she criticize you or your ideas				
How often does she help you to do things that are important to you				
How often does she make plans with you and cancel for no reason				

23. Do you share your personal information with your mother?

a) Yes, always

b) Most of the time

c) Sometimes

d) Never

24. Carefully read the below statements and state True (T) or False (F) in relation to your mother. When having a conversation with my mother;

	True	False
a) I am often criticized, when I'm discussing things with my mother		
b) My mother blames me for everything		
c) My mother talks to me in a superior way		
d) I don't think my mother respects me		
e) My mother will not have long discussions with me		
f) My mother refuses to talk about some topics		
g) My mother won't listen to what I want to say		
h) When we talk, my mother is defensive		
i) My mother's feelings are too easily hurt		
j) I can talk to my mother about things that are important to me		

Mother-adolescent communication of sexual health

25. Do you think it's a good decision to discuss with your mother regarding sex related issues? (e.g. pregnancy, contraception, abortion)?

a) Yes b) No

If your answer is 'No' to the above question, go to Q25. If your answer is 'Yes' go to question Q26

26. Why do you think it's not a good decision to discuss with your mother regarding sex related issues? (Please put a '√' in the correct box given)

a) It's embarrassing to discuss about 'sex' with parents ☐

b) I'm scared. They might misunderstand me and think I'm evil. ☐

c) My mother does not have time for an open discussion with me. ☐

d) I don't think my mother has the ability to answer my questions. ☐

e) I have lot more ways to find information other than my parents. ☐

27. Do you think your mother is capable of answering your questions regarding sexual health?

a) Yes b) No

28. Have your mother ever talked to you anything related to below mentioned topics? (please put a ‘x’ in the correct box). Girls please fill Table A. Boys fill Table B.

A.

Topics	Have you ever discussed?	
	Yes	No
a) Issues related to Menstrual cycle		
b) Consequences of pre-marital sex		
c) Consequences of Early marriages		
d) Avoiding unwanted pregnancy		
e) Homosexuality		
f) Preventing from Sexually Transmitted diseases including HIV& AIDs		
g) Preventing Sexual violence and abuse		

29. Do you think discussing with your parent on above topics can help you to face certain sexual issues that you might face in future?

a) Yes b) No c) Do not know

30. If you have already discussed with your parent on above topics, are you satisfied with the answers you got from your parents for your questions?

a) Yes b) No c) Don’t know

31. Has your mother ever talked to you regarding sexual abuse?

a) Yes b) No c) Not sure

32. Has your mother ever explained to you about your personal boundaries (e.g. If anyone tries to touch your body or do something that makes you uncomfortable, tell them ‘No’ and tell me right away)

a) Yes b) No c) Not Sure

Adolescent perceived barriers for discussing sexual and reproductive information with their mothers

33. If you have not discussed with your parents so far on Sexual and Reproductive Health (SRH) issues what are the reasons for not discussing? (Please mark a ‘√’ in the correct box given)

a) Talking about sex is culturally not acceptable
b) I feel embarrassed to discuss these topics with my parents
c) I don’t think my parents have knowledge to answer my questions on these topics
d) My parents won’t discuss with me about sex
e) My parents are not good listeners. They will not understand me.

f) My parents are too busy. They don’t have time to communicate with me on these matters.
g) I don’t know why I don’t discuss with my parents on these topics.

34. What do you expect from your mother in order to share your personal information.

	Yes	No	Not sure
a) I want my mother to be closer to me and approachable			
b) I want my mother to be more knowledgeable regarding adolescent sexual matter			
c) I want her to spend more time with me so I can come up with my issues			
d) I want her to assure the privacy and confidentiality of the things I tell her			
e) I want her to have more faith in me and believe in what I tell her			

Common sources of sexual health information

35. What are your common sources of sexual health information? (Mark a ‘√ ‘ in front of the correct answers)
    a. Television or radio
    b. School
    c. Father
    d. Mother
    e. Siblings
    f. Friends
    g. Books and Magazines
    h. Internet
    i. Other

36. What are your preferred sources of sexual health information? (Mark a ‘x ‘ in front for the correct answers)
    a. TV or radio
    b. School
    c. Father
    d. Mother
    e. Siblings
    f. Fiends
    g. Books or magazines

h. Internet

i. Others
