# Supplementary material for: Mother-daughter communication of sexual and reproductive health (SRH) matters and associated factors among sinhalese adolescent girls aged 14–19 years, in Sri Lanka
Source: BMC Womens Health. 2023 Aug 31;23:461. doi: 10.1186/s12905-023-02617-4 (PMC10472576; doi:10.1186/s12905-023-02617-4)
Supplement: Supplementary file 2 — Additional File 2: About the topic [file 12905_2023_2617_MOESM2_ESM.docx]

**What is already known about this topic?**

Studies that have been carried out in Sri Lanka so far have indicated that a majority of adolescents contacted their friends for sexual health matters. Although some adolescents would turn to their parents for minor sexual health matters, they avoid discussing most of the sexual health topics with their parents.

However, studies that have been carried out across the world have shown that family-based sexuality education has been effective in improving adolescent’s knowledge, attitudes and practices on SRH. In order to test such interventions in Sri Lanka, it is essential to assess the current practice of mother-daughter SRH communication in the study setting.

**What this study adds?**

This study will explore the potential for family-based sexuality education of adolescents in the study setting. It will discover adolescents’ perception of communicating SRH matters with their mothers.

**How this study might affect the research, practice, or policy?**

This study will give information to initiate family-based interventions to improve adolescent girls’ knowledge, attitudes and practices on SRH.
